# PROTOCOL: Conflict of interest issues when engaging stakeholders in health and healthcare guideline development: a systematic review

**DOI:** 10.1002/cl2.1232

**Published:** 2022-04-16

**Authors:** Joanne Khabsa, Jennifer Petkovic, Alison Riddle, Lyubov Lytvyn, Olivia Magwood, Pearl Atwere, Pauline Campbell, Srinivasa V. Katikireddi, Bronwen Merner, Mona Nasser, Stephanie Chang, Alejandra Jaramillo Garcia, Heather Limburg, Jeanne‐Marie Guise, Peter Tugwell, Elie A. Akl

**Affiliations:** ^1^ Clinical Research Institute American University of Beirut Medical Center Beirut Lebanon; ^2^ Bruyère Research Institute University of Ottawa Ottawa Canada; ^3^ School of Epidemiology and Public Health, Faculty of Medicine University of Ottawa Ottawa Canada; ^4^ Department of Clinical Epidemiology and Biostatistics McMaster University Hamilton Canada; ^5^ C.T. Lamont Primary Health Care Research Centre Bruyère Research Institute Ottawa Canada; ^6^ Nursing, Midwifery and Allied Health Professions Research Unit Glasgow Caledonian University Glasgow UK; ^7^ MRC/CSO Social and Public Health Sciences Unit University of Glasgow Glasgow UK; ^8^ School of Psychology and Public Health, Centre for Health Communication and Participation La Trobe University Bundoora Australia; ^9^ Peninsula Dental School Plymouth University Peninsula Schools of Medicine and Dentistry Plymouth UK; ^10^ Annals of Internal Medicine American College of Physicians Washington, DC USA; ^11^ Applied Research Division Centre for Surveillance and Applied Research Health Promotion and Chronic Disease Prevention Branch, Public Health Agency of Canada/Government of Canada Ottawa Canada; ^12^ Global Health and Guidelines Division Public Health Agency of Canada Ottawa Canada; ^13^ Departments of Obstetrics and Gynecology, Medical Informatics and Clinical Epidemiology, Public Health & Preventive Medicine, and Emergency Medicine Oregon Health and Science University Portland Oregon USA; ^14^ Department of Medicine, Faculty of Medicine University of Ottawa Ottawa Canada; ^15^ Department of Internal Medicine American University of Beirut Beirut Lebanon; ^16^ Department of Health Research Methods, Evidence, and Impact (HEI) McMaster University Hamilton Ontario Canada

## Abstract

This is the protocol for a Campbell systematic review. The overall objective of this study is to gather and summarize the existing literature on conflict of interest issues when engaging stakeholders in guideline development.

## 1 BACKGROUND

### 1.1 The problem, condition, or issue

Stakeholder engagement in research helps to ensure that research is relevant to, and used by those affected by it (CIHR, 2012). Similarly, a growing importance is being placed on the engagement of stakeholders in the development of guidelines (Rosenfeld et al., 2013). Schünemann et al. (2014) reviewed guideline development manuals as well as methodology reports and identified stakeholder engagement as a distinct step in the guideline development process (Schünemann et al., 2014). Accordingly, organizations that develop practice guidelines are designing programs that promote user and public participation (Boivin et al., 2010).

Stakeholders are defined as “an individual or a group who is responsible for or affected by health‐ and healthcare‐related decisions” (Concannon et al., 2012; Tugwell et al., 2006). Hence, they implicitly have interests or “stakes” in the outcome of a guideline project. Stakeholders could have other interests that conflict with the primary interests of the guideline project. For example, two studies examining patient representatives at the United States Food and Drug Administration (USFDA) regulatory processes found that patient representatives had personal conflicts of interest (COI) emanating from the receipt of fees from the industry as well as COI resulting from their organizations' ties to pharmaceutical companies (Abola & Prasad, 2016; R. Graham et al., 2011). Also, an observational study of 713 US journal editors found that 51% received general payments and 20% received research payments from the industry in 2014 (Liu et al., 2017).

There is evidence that authors' financial, intellectual and professional conflicts may influence guideline recommendations (Norris et al., 2011, 2012a). For instance, recommendations favouring routine screening for breast cancer were associated with lead authors' speciality (e.g., radiology), and having a higher number of recent publications on breast cancer diagnosis and treatment (Norris et al., 2012a).

When considering stakeholder groups, it might be at times appropriate to fully include them, while at other times it might be appropriate to only partially include them, or not include them at all (e.g., private sector representatives). Therefore, stakeholder engagement requires fair and transparent disclosure, management, and reporting of COI.

### 1.2 Definitions

We will adopt the following definitions

- Guidelines: “systematically developed evidence‐based statements which assist providers, recipients and other stakeholders to make informed decisions about appropriate health interventions” (World Health Organization, 2003, p. 2).
- Stakeholders: an individual or a group who is responsible for or affected by health‐ and healthcare‐related decisions (Concannon et al., 2012; Tugwell et al., 2006).
- Engagement: approach to ensure the contribution of stakeholders toward the development of the guideline, completion of any of the stages of the guideline, or dissemination of the guideline and its recommendations, adapted from Pollock et al. (2018). Terms such as involvement, collaboration, or partnership have also been used to refer to engagement (Hoddinott et al., 2018). Within this review we will use the term “engagement.”
- COI: “a set of circumstances that creates a risk that professional judgment or actions regarding a primary interest will be unduly influenced by a secondary interest" (Lo & Field, 2009), by the Institute of Medicine (IOM).
- COI policy: any statement that provides “information required for disclosure of financial and nonfinancial relationships” or “procedures for collecting, reviewing disclosure of relationships of interest and managing conflict of interest" (Morciano et al., 2016).

### 1.3 Why it is important to do the review

The potential for COI among stakeholders is most frequently dealt with in the research setting (Concannon et al., 2012, 2014; Guise et al., 2013; O'Haire et al., 2011). In the setting of guideline development, the issue of COI is often not addressed in the context of stakeholder engagement. In fact, studies examining COI in guideline development often focus solely on experts (Khan et al., 2018; Neuman et al., 2011), with other stakeholders less studied. This is despite COI being common amongst stakeholders, such as patient groups (Khabsa et al., 2019), which are increasingly influential in guideline development. Guideline authors were found to have financial COI, such as the receipt of payments for advising and consulting, research grants, and stocks; as well as nonfinancial COI, such as academic activities (Norris et al., 2012b). However, there is uncertainty about the types of COI that stakeholders engaged in the guideline development process could have, and their prevalence.

There are also uncertainties regarding the approaches for addressing COI of stakeholders in guideline development, including COI disclosure, management and reporting. For example, Armstrong et al. (2017) provide a 10‐step framework for “patient” engagement in clinical practice guidelines. The study does not provide specific guidance on how to address COI issues among patients themselves. However, as part of the step “selecting guideline development group members” the authors suggest that patients assess the COI of panel members, from their perspective (Armstrong et al., 2017).

Schünemann et al. (2015) discuss how patients may be subject to COI if they are given support from organizations that are funded by the industry. They also highlight how policy makers' interests, that include pleasing the public on one hand or losing their professional standing on the other, can interfere in their ability to remain objective in the guideline development process. Authors propose nine principles for disclosing and managing interests, which address the guideline development group in general. According to the authors, the definition of COI and its management applies to all members of a guideline development group. None of the principles was specific to patients, consumers, or other stakeholders (Schünemann et al., 2015).

Finally, evidence from other settings suggests that COI influence the guideline product. For example, patients acting as public speakers at drug regulatory processes and disclosing COI were at higher odds of supporting drug approval than those who did not (McCoy et al., 2018). Jørgensen et al. reported an association between funding of patients' organizations by the pharmaceutical industry and presentation of information about breast cancer screening on the organizations' websites (Jørgensen & Gøtzsche, 2004). Similarly to the research field where COI could affect public trust (Cigarroa et al., 2018), there are concerns regarding the effect of COI of stakeholders on the trustworthiness and accountability of the guideline development process and on its output. Indeed, the IOM considers establishing COI policies and implementing them to be important aspects of accountability (Lo & Field, 2009).

In 2015, we established the Multi Stakeholder Engagement (MuSE) working group (Frank et al., 2019). The MuSE working group is a global consortium of researchers and stakeholders from various countries including: Australia, Canada, Germany, Italy, Lebanon, the Netherlands, the Philippines, Switzerland, the United Kingdom and United States. The group includes representatives from universities, the Agency for Healthcare Research and Quality (AHRQ), the Campbell Collaboration, Cochrane, Grading of Recommendations Assessment, Development and Evaluation (GRADE) Working Group, Health Canada, the Patient‐Centered Outcomes Research Institute (PCORI), Research ANd Development (RAND) Corporation, and the World Health Organization (WHO). All group members share an interest in developing methods and approaches for involving patients and other stakeholders in health outcomes research (Concannon et al., 2012). Our collaboration identified the need for new tools, resources and guidance on stakeholder engagement.

This review is part of a series of four linked reviews conducted by the MuSE working group to develop guidance for multi‐stakeholder engagement in guideline development (Petkovic et al., 2020). These reviews deal with different aspects of stakeholder involvement including:

- Existing guidance: the objective of this review is to synthesize existing guidance for stakeholder engagement at each of the 18 steps in the guideline development process.
- Barriers and facilitators: the objective of this review is to summarize the barriers and facilitators to stakeholder engagement at each step in the guideline development process.
- COI (current study).
- Impact: the objective of this review is to assess the impact of stakeholder engagement in guideline development on (a) the guideline development process, (b) guideline relevance, trustworthiness, acceptability, and uptake, and (c) the stakeholders and panel members themselves.

The results of this review will inform the development of GRADE Working Group guidance for multi‐stakeholder engagement in guideline development. The GRADE system is an internationally recognized standard for guideline development (Guyatt et al., 2008). Currently, the GRADE Handbook states that “the guideline panel and supporting groups […] work collaboratively, informed through consumer and stakeholder involvement” but does not provide specific guidance on how this should be achieved (Schünemann et al., 2013).

Many guideline development organizations provide guidance for guideline development but do not provide specific information about how and when to engage multiple stakeholders. Our review will help to fill this gap by addressing COI issues when engaging stakeholders in the guideline development process. The findings of this review, along with the other three in the series, will assist organizations who develop healthcare, public health, and health policy guidelines, such as the WHO, to involve multiple stakeholders in the guideline development process to ensure the development of relevant, high quality, and transparent guidelines.

## 2 OBJECTIVES

The overall objective of this study is to gather and summarize the existing literature on conflict of interest issues when engaging stakeholders in guideline development. Specifically, this review will answer the following questions:

1. What are the types and prevalence of relevant interests and COI amongst stakeholders engaged in the guideline development process, and how do they vary by stakeholder group?
2. How do the COI of stakeholders impact the guideline development process, the produced recommendations, and end‐users' perceptions?
3. What are the proposed and/or implemented approaches for disclosing, managing and reporting the COI of stakeholders engaged in guideline development?
4. What are the COI considerations related to the process of selecting stakeholders to participate in guideline development?

## 3 METHODS

### 3.1 Criteria for including and excluding studies

#### 3.1.1 Types of study designs

Our methods will follow the Cochrane Handbook for Systematic Reviews of Interventions and the Handbook for Synthesizing Qualitative Research as appropriate (Higgins et al., 2019; Sandelowski & Barroso, 2006). We will include randomized trials, non‐randomized studies (e.g., cohort studies, controlled and noncontrolled before and after studies, cross‐sectional studies), qualitative studies, process evaluation studies, policy analysis studies, case studies, and mixed methods studies. We will exclude editorials, commentaries, proposals, study protocols and conference abstracts. Study design eligibility is the same across all review objectives.

We will include studies addressing the COI of stakeholders in guideline development. As such, we will exclude handbooks or manuals for guideline development by guideline producing organizations. We assess these in a separate publication that will include only handbooks.

Mixed methods studies that apply a combination of the eligible quantitative and qualitative study designs and report on qualitative and quantitative outcomes separately will be eligible.

#### 3.1.2 Types of participants

For this review, “participants” are the groups of stakeholders considered.

We have identified 13 types of stakeholders whose input can enhance the relevance and uptake of research (Concannon, 2012, 2019; Tugwell et al., 2006); as follows:

- patients, caregivers, and patient advocates;
- the public;
- providers of health care;
- payers of health services;
- payers of research;
- policy makers;
- program managers;
- product makers;
- purchasers;
- principal investigators and their research teams; and
- peer‐review editors (i.e., editors of peer‐reviewed publications).

This review will include all the above types of stakeholders except for principal investigators. We will also include studies addressing stakeholder groups that are not included in the above list.

#### 3.1.3 Types of outcome measures

Outcomes of interest by research question:

- Research question 1: type and prevalence of relevant interests and COI by stakeholder group.
- Research question 2: impact of COI on the guideline process; impact of COI on the produced recommendations; impact of COI on end‐users' perceptions (e.g., trust).
- Research question 3: approaches for the disclosure of COI of stakeholders; approaches for the management of the COI of stakeholders; approaches for the reporting of the COI of stakeholders.
- Research question 4: impact of COI on the process of selecting stakeholders.

#### 3.1.4 Types of settings

We will include studies discussing any step of the guideline development process, as described by the GIN‐McMaster Guideline Development Checklist (Schünemann et al., 2014, fig. 1), in any setting. We will restrict eligibility to health and healthcare‐related guidelines. We will exclude guidelines where health is not the primary focus (e.g., climate change guidelines).

### 3.2 Search strategy

As mentioned above, this review is part of a series of four reviews conducted by the MuSE working group on stakeholder engagement in guideline development. As such, we will develop one comprehensive search strategy in consultation with a medical librarian which will be reviewed by a second medical librarian. We will search the following databases: MEDLINE (OVID), CINAHL (EBSCO), EMBASE (OVID), PsycInfo (OVID), SCOPUS, and Sociological Abstracts (Supporting Information Appendix 1). We will not place limits on language, date, or study design. In addition, we will perform both backward and forward citation tracking to identify further eligible studies.

To identify gray literature, we will search the websites of agencies who actively engage stakeholder groups such as the AHRQ, Canadian Institutes of Health Research (CIHR) Strategy for Patient‐Oriented Research (SPOR), INVOLVE, the National Institute for Health and Care Excellence (NICE), and the PCORI. We will also search the websites of guideline‐producing agencies, such as the American Academy of Pediatrics, Australia's National Health Medical Research Council (NHMRC), and the WHO including Latin American and Caribbean Health Sciences Literature (LILACS).

We will invite members of the MuSE working group to suggest gray literature sources and we plan to broaden the search by soliciting suggestions via social media, such as Twitter.

### 3.3 Description of methods used in primary research

We anticipate included studies to include methodological studies and frameworks, cross‐sectional study designs (e.g., surveys), and qualitative study designs. An example of a typical study addressing objective 1 would be a qualitative study exploring the nature of COI among stakeholders of interest, in the context of their engagement in guideline development. Atkins et al. interviewed a purposive sample of 39 members of three NICE advisory groups. The interviews focused on the step “how evidence is translated into recommendations.” Participants perceived that group members would bring their own “vested interests” to the table, influenced by their experiences, their academic or professional speciality, or allegiance to particular products (e.g., types of treatment).

### 3.4 Criteria for determination of independent findings

If eligible information is included in more than one report of the same study, we will gather relevant information from all reports. When more than one report addresses the same outcome, we will refer to the most recent one. We will use information on study sample sizes, guideline details, grant numbers, and so on to judge whether different reports relate to the same study. If needed, we will contact the authors of the reports for any needed clarification.

### 3.5 Data collection and analysis

Teams of two reviewers will screen titles and abstracts independently and in duplicate to identify relevant studies meeting the pre‐specified inclusion criteria. Similarly, the full text of potentially included studies will be screened independently and in duplicate by teams of two reviewers. We will use Covidence software (https://www.covidence.org/) for screening of studies.

We will extract data on study general characteristics (e.g., year of publication, study design, study aim), steps of the guideline development process addressed (Schünemann et al., 2014, fig. 1), types of stakeholders involved (i.e. above categorization), the health area of interest when relevant, and outcomes of interest as listed above for the four objectives. We will also extract data on the level of stakeholder engagement in included studies. We identify two levels of engagement namely (1) advisory/feedback, and (2) participating in decision‐making and/or knowledge translation. We will also abstract data on other levels of engagement if any.

We will critically appraise the included studies using appropriate tools. We will use the Cochrane risk of bias tool for randomized trials, the Risk of Bias in Non‐randomized Studies—of Interventions (ROBINS‐I) tool (Sterne et al., 2016) for non‐randomized studies of interventions, NOS for non‐randomized studies of exposures, and the Critical Appraisal Skills Programme (CASP) qualitative appraisal research tool (CASP, 2018) for qualitative studies. We will use the Mixed Methods Appraisal Tool (MMAT) to assess the quality of mixed‐methods studies (Hong et al., 2018). Critical appraisal will be conducted independently and in duplicate by two authors and any discrepancies will be resolved by consensus and consultation with a third author, when necessary.

We will develop and pilot test a standardized data abstraction sheet with detailed instructions. Two reviewers will extract data independently and in duplicate using a structured Excel sheet. The sheet will be piloted on ten articles. Disagreements on extractions will be resolved by discussion and with a third member of the research team when necessary.

### 3.6 Data synthesis

We will analyze continuous variables using mean and 95% confidence intervals when data are normally distributed; otherwise, we will use the median and interquartile range (IQR). We will describe categorical variables using frequencies and percentages using Excel. We will summarize the findings in both narrative and tabular formats. We will aim to conduct stratified analysis by the level of engagement and by the step of the guideline development process when enough data is available.

### 3.7 Treatment of qualitative research

We will follow the guidance for thematic synthesis of qualitative research in systematic reviews as outlined by Thomas and Harden (2008), which includes coding the text and developing descriptive themes (Thomas & Harden, 2008). Abstracted data will include participant quotations from interviews or focus groups, narrative descriptive summaries, author hypotheses, explanations and recommendations, themes and sub‐themes. We expect qualitative data to address perceptions, experiences or concerns related to COI of stakeholders and their types. Depending on the included studies, general categorizations of COI (e.g., financial and nonfinancial COI; personal and institutional COI), or other frameworks can be used to guide the coding scheme and thematic synthesis. When COI issues are reported at specific steps of the guideline development process, we will refer to the GIN‐McMaster Guideline Development Checklist (Schünemann et al., 2014). We will translate concepts from one study to another, and group concepts into themes for analysis by looking for similarities and differences between codes (Thomas & Harden, 2008). Each step will be discussed amongst members of the review team. If applicable, we will use the Confidence in the Evidence from Reviews of Qualitative research (CERQual) tool to assess the confidence of our findings (Lewin et al., 2015). If we identify a large number of qualitative studies, we will consider sampling from the included studies according to the EPOC guidance (EPOC, 2021). Sampling will be based on the following key elements: objective answered by the study, types of stakeholders covered, step of the guideline development process examined, level of engagement, and data richness.

## CONTRIBUTIONS OF AUTHORS

- Writing the protocol: EAA, JK, JP, AR, LL, PA, OM, PC, SVK, BM, MN, SC, AJG, HL, JMG
- Coordinating the review: JK, EAA
- Systematic review methods: EAA, PT, JP, AR, LL, JK

## DECLARATIONS OF INTEREST

The views of the authors are not influenced by the Canadian Institutes of Health Research (CIHR). SVK acknowledges funding from a NRS Senior Clinical Fellowship (SCAF/15/02), the Medical Research Council (MC_UU_00022/2) and the Scottish Government Chief Scientist Office (SPHSU17). EAA is an author/contributor to multiple studies on the topic of conflicts of interest which have the potential to be included in this review. The rest of the authors declare no conflict of interest.

## SOURCES OF SUPPORT

**External sources**

- Canadian Institutes of Health Research, Canada

Funding (PJT‐155970; Application number: 391110)

## Supporting information

Supporting information.
